# Engrailed 1 shapes the dopaminergic and serotonergic landscape through proper isthmic organizer maintenance and function

**DOI:** 10.1242/bio.015032

**Published:** 2016-02-15

**Authors:** Willemieke M. Kouwenhoven, Jesse V. Veenvliet, Johannes A. van Hooft, L. P. van der Heide, Marten P. Smidt

**Affiliations:** Swammerdam Institute for Life Sciences, University of Amsterdam, P.O. Box 94215, 1090 GE Amsterdam, The Netherlands

**Keywords:** mdDA, Engrailed 1, IsO, Mouse, Patterning

## Abstract

The isthmic organizer (IsO) is a signaling center that specifies the correct and distinct embryonic development of the dopaminergic midbrain and serotonergic hindbrain. The IsO is a linear boundary between the two brain regions, emerging at around embryonic day 7-8 of murine embryonic development, that shapes its surroundings through the expression of instructive signals such as Wnt and growth factors. Homeobox transcription factor engrailed 1 (En1) is present in midbrain and rostral hindbrain (i.e. rhombomere 1, R1). Its expression spans the IsO, and it is known to be an important survival factor for both dopaminergic and serotonergic neurons. Erroneous composition of dopaminergic neurons in the midbrain or serotonergic neurons in the hindbrain is associated with severe pathologies such as Parkinson's disease, depression or autism. Here we investigated the role of En1 in early mid-hindbrain development, using multiple En1-ablated mouse models as well as lineage-tracing techniques, and observed the appearance of ectopic dopaminergic neurons, indistinguishable from midbrain dopaminergic neurons based on molecular profile and intrinsic electrophysiological properties. We propose that this change is the direct result of a caudal relocation of the IsO as represented by ectopic presence of Fgf8, Otx2, Wnt1 and canonical Wnt-signalling. Our work suggests a newly-discovered role for En1: the repression of Otx2, Wnt1 and canonical Wnt-signaling in R1. Overall, our results suggest that En1 is essential for proper IsO maintenance and function.

## Abstract (precis)

**Summary:** Local molecular coding under the influence of En1 is essential for proper spatiotemporal expression of key factors involved in the maintenance and function of the isthmic organizer.

## INTRODUCTION

The correct and distinct embryonic development of the midbrain and hindbrain is specified by a signaling center between these brain areas: the isthmic organizer (IsO). The mid- and hindbrain region harbor two essential neurotransmitter systems: mesodiencephalic dopaminergic (mdDA) neurons, which express a plethora of dopaminergic markers such as Th, Pitx3, Nurr1, Otx2, En1, En2, Dat, Lmx1a/b, Vmat2, Aadc, Pbx1, Pbx3, (Smidt and Burbach, 2007), and serotonergic (5-HT) neurons which express different markers such as Sert, Pet1 and Gata3 (Deneris and Wyler, 2012; Smidt and van Hooft, 2013). The correct function of the IsO is essential in determining the location and respective size of these neuronal systems.

The IsO is formed at around embryonic day (E)7-8 of murine embryonic development, at the exact position where the expression of mesencephalic Otx2 and metencephalic Gbx2 meet (Martinez-Barbera et al., 2001; Millet et al., 1999; Wassarman et al., 1997). Through the expression of fibroblast growth factor 8 (Fgf8) (Crossley and Martin, 1995) and secreted glycoprotein Wnt1 (Wilkinson et al., 1987), the IsO provides the surrounding areas with instructive signals that lead to the induction of mdDA neurons located in midbrain and 5HT neurons in the hindbrain (Brodski et al., 2003; Ye et al., 1998). Disruption of the Otx2/Gbx2 boundary results in a positional shift of the IsO, and this is accompanied by an enlarged or reduced mid- or hindbrain (Broccoli et al., 1999; Martinez et al., 1999; Millet et al., 1999; Wassarman et al., 1997).

The homeobox transcription factor engrailed 1 (En1) is involved in development and maintenance of the monoaminergic structures in the mid- and hindbrain (Deneris and Wyler, 2012; Fox and Deneris, 2012; Jensen et al., 2008; Lundell et al., 1996; Simon et al., 2005; Smidt and Burbach, 2007; Smidt and van Hooft, 2013; Wylie et al., 2010), and has been implicated to contribute to the maintenance of the IsO in zebrafish (Scholpp et al., 2003). Previous studies on the developmental role of En1 in the murine mid- and hindbrain region has long been hindered due to the assumption that absence of the En1 alleles results in the complete ablation of the cerebellum with perinatal lethality (Wurst et al., 1994). However, our group and others recently showed that this cerebellar ablation and perinatal lethality is caused by the genetic background of the mouse strain. This phenotype can be circumvented by back crossing the original 129/Sv line to a C57BL/6J background (Bilovocky et al., 2003; Veenvliet et al., 2013). Using this approach we showed that in the absence of En1, the expression of mdDA markers is diminished during embryogenesis. Paradoxically, ectopic mdDA markers can be detected in the metencephalon (Veenvliet et al., 2013). Furthermore, in a genome wide expression analysis of En1KO animals it was shown that besides the dopaminergic markers also several markers that are associated with the serotonergic system were down regulated, such as Gata3, Penk1 (Veenvliet et al., 2013). The ectopic expression of dopaminergic markers in the metencephalon and the deregulated serotonergic markers in the genome wide expression analysis suggest that En1 influences the cytoarchitecture of midbrain and the rostral hindbrain. In the present study we show that the dopaminergic neurons in the metencephalon are molecularly indistinguishable from correct positioned midbrain dopaminergic neurons. All critical dopaminergic markers that are present in the mdDA neurons of En1-ablated animals (*Pitx3*, *Nurr1*, *Lmx1b*, *En2*, *Otx2*, *Th*, *Dat*, *Vmat2*, *Aadc*, *Pbx1* and *Pbx3*) are also present in the ectopic Th-expressing cells. Furthermore, electrophysiological recordings indicate that the ectopic Th-expressing cells fully resemble control mdDA neurons, both at prenatal and postnatal stages. In line with this observation, we show that the expansion of the DA neuronal field is accompanied by a diminished amount of 5-HT neurons in rhombomere 1 (R1). We propose that the appearance of ectopic dopaminergic (eDA) neurons is the direct result of an extension of midbrain patterning in R1, due to a caudal relocation of the IsO as represented by ectopic presence of Fgf8, Otx2, Wnt1 and canonical Wnt-signaling. In conclusion, En1 is essential for proper IsO maintenance and function.

## RESULTS

### Ectopic dopaminergic neurons arise in rostral hindbrain in absence of En1

**Fig. 1.**
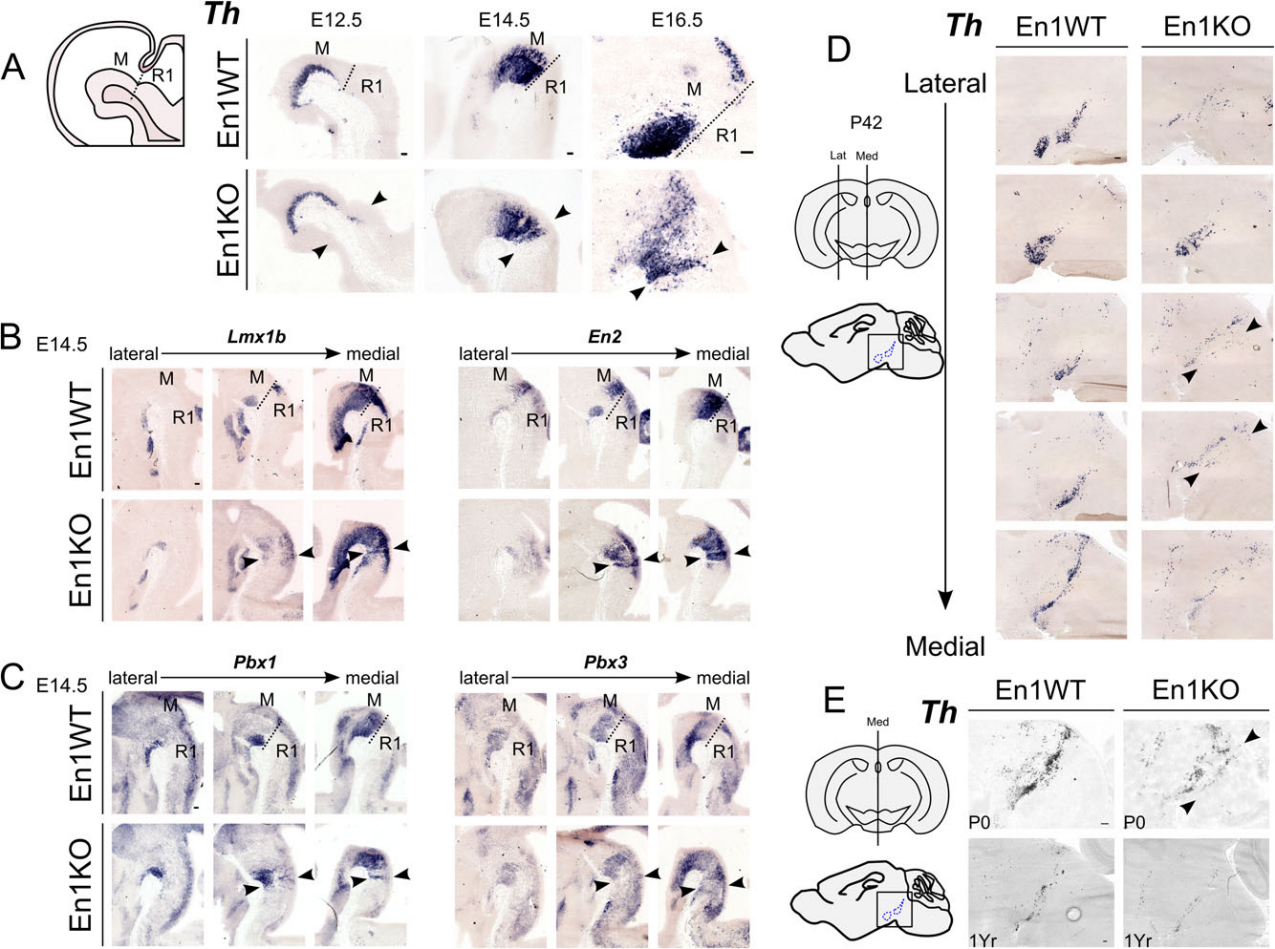
**Ectopic dopaminergic neurons are found in the hindbrain of the En1KO as early as E12.5 and remain present into adulthood.** (A) *Th* expression is found in the rostral hindbrain of En1 knockout embryos (En1KO) at prenatal stages embryonic day (E)12.5, E14.5 and E16.5 (arrowheads), as compared to wild-type embryos (En1WT). (B,C) Earlier dopaminergic markers, *Lmx1b* and *En2* (B), and transcription factors that are involved in DA subset specification like *Pbx1* and *Pbx3* (C) are ectopically present in absence of En1 at E14.5 (arrowheads). (D,E) Sagittal sections of neonate and adult En1KO midbrain and hindbrain at postnatal day (P)0 and P42, reveal *Th* transcript is present in hindbrain (arrowheads). Both the size of the midbrain and ectopic Th-population seems to progressively diminish, as a consequence of the En1-ablation (Veenvliet et al., 2013). At 1 year it is difficult to differentiate between mdDA and eDA neurons. (Para)medial sections as shown in schematic: M, midbrain; R1, rhombomere 1; dotted line represents the position of the isthmus. Scale bars: 100 μm.

To further substantiate our initial observations at E14.5 that in absence of En1 cells emerge in the metencephalon that possess a dopaminergic profile (expressing *Pitx3*, *Nurr1*, *Th*, *Dat*, *Vmat2*, *Aadc*; Fig. S1) (Veenvliet et al., 2013) we mapped the appearance of the ectopic DA neurons in time (Fig. 1A). Similarly we investigated the presence of essential transcription factors, expressed during early DA development (before E11) such as *Lmx1b* and *En2* (Smidt and Burbach, 2007; Smidt et al., 2000a), and transcription factors *Pbx1* and *Pbx3*, that are involved in DA subset specification (Veenvliet et al., 2013). As early as E12.5 *Th* expression can be detected in an ectopic location caudal to the midbrain (Fig. 1A, arrowheads), indicating that at the first sign of dopaminergic development the ectopic DA neurons arise and these neurons can still be detected later in development at E16.5.

In En1KO animals, *Lmx1b* expression is enhanced in the ventral hindbrain area, which is especially apparent in more medial positions (Fig. 1B, middle panel, arrowheads). Furthermore, the *En2* expression is extended into the hindbrain (Fig. 1B, right panel, arrowheads). Moreover, subset markers *Pbx1* and *Pbx3* are both ectopically expressed in the (ventral) hindbrain. Notably, the expression pattern of *Pbx1* matches the ectopic expression of *Th* at E14.5 (Fig. 1C, arrowheads).

To assess whether these initial developmental defects are lasting towards adulthood we analyzed sagittal sections of neonatal and adult En1KO brains. Ectopic DA (eDA) neurons, identified by the expression of *Th*, are still found in (para)medial sections of the neonatal and adult En1KO brain at postnatal day (P)0 and P42 (Fig. 1D,E, arrowhead). Note however, that at P42 the density *Th*-positive neurons was somewhat diminished in SN and VTA as a consequence of the En1-ablation (Veenvliet et al., 2013). This similar loss of cell density was observed for the *Th*-expressing cells in the metencephalon (compare prenatal stages with P0 and P42, Fig. 1). At 1 year after birth only a few *Th*-positive cells are still present in the En1KO, probably due to earlier described progressive degeneration in the absence of En1 (Veenvliet et al., 2013). Consequently, while it is no longer possible to clearly determine the exact location of the small amount of remaining *Th* neurons, it suggests that the eDA neurons are also characterized by a progressive cell loss possibly similar to mdDA neurons in the absence of En1. Taken together, the analysis from embryonic, neonatal and adult En1KO material indicates that eDA neurons in En1KO mice are molecularly similar to mdDA neurons in these mice.

### Ectopic DA neurons are indistinguishable from mdDA neurons based on their intrinsic electrophysiological properties

**Fig. 2.**
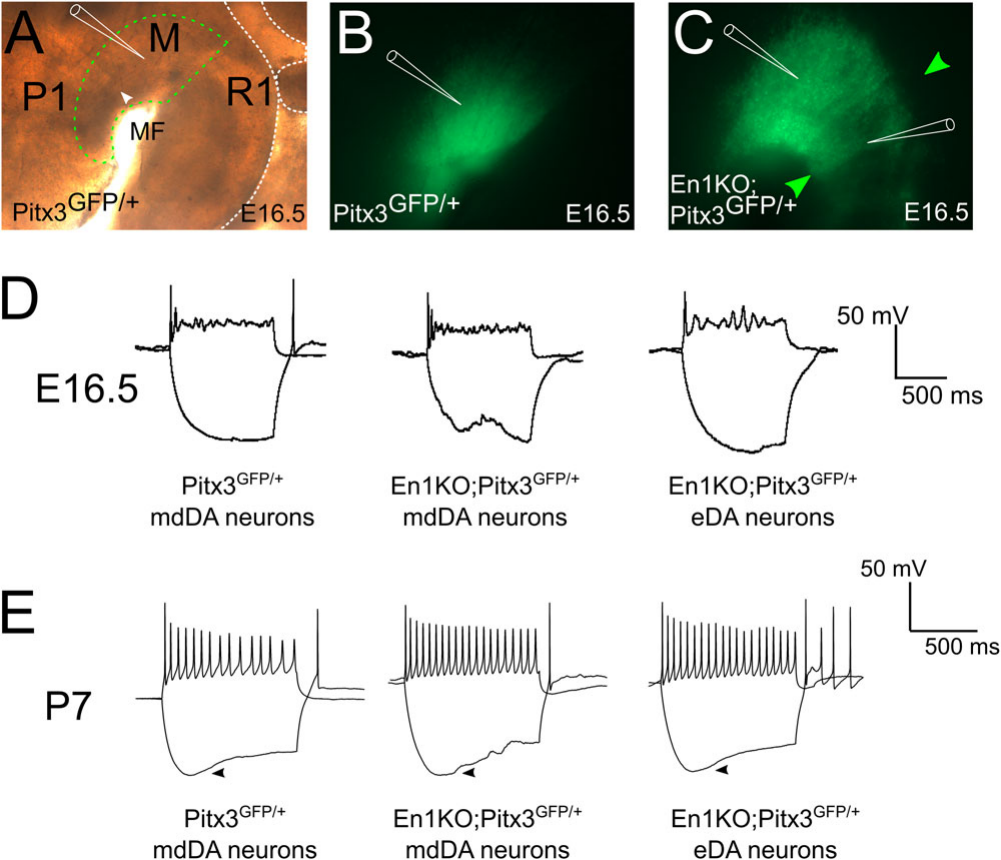
**eDA neurons are electrophysiologically identical to control mdDA neurons.** (A) Bright field image of a medial plane of a hemitube at E16.5, including a demarcation of mdDA area by a green dotted line. P1, prosomere 1; M, midbrain; R1, rhombomere 1; MF, mesencephalic flexure. White arrowhead shows the location of the retroflexus. The white dotted line marks the outline of the mesencephalic tissue. (B,C) Visualization of mdDA area by endogenous Pitx3GFP signal in control (B) and *En1KO;Pitx3GFP/+* (C) animals, including eDA neurons (green arrowheads) in hemitubes at E16.5. Schematic electrodes in A-C indicate locations of recordings. (D,E) Traces at E16.5 (D) and P7 (E) exemplify the absence of dissimilarities between control mdDA neurons, *En1KO;GFP/+* mdDA neurons and *En1KO;GFP/+* eDA neurons. The presence of the hyperpolarizing sag reveals the dopaminergic characteristic of the h-current in all three conditions at P7 (arrowhead).

To further proof that the observed eDA neurons are (functionally) similar to mdDA neurons we investigated their electrophysiological profile at different developmental stages. In order to specifically visualize mdDA neurons in living slices we elected to use *Pitx3GFP/+* mice, in which GFP is uniquely expressed under the control of Pitx3 (Maxwell et al., 2005). Since we showed that Pitx3 is present in eDA neurons (Fig. S1), we inter crossed En1 mutant animals with *Pitx3GFP/+* animals, ultimately generating *En1KO;Pitx3GFP/+* animals, thus introducing GFP expression in mdDA neurons and eDA neurons (Fig. 2A-C, Fig. S2).

Recordings from (a) midbrain *Pitx3GFP/+* neurons, (b) *En1KO;Pitx3GFP/+* midbrain neurons and (c) eDA neurons in *En1KO;GFP/+* animals at E16.5 revealed a high input resistance and a depolarized resting membrane potential within all three conditions, and a general absence of spontaneous action potential firing (Fig. 2D, Table 1). This is indicative of the immature state of these neurons at E16.5, and based of the passive electrophysiological properties, this state was not different between the three groups (*P*>0.05 between the three conditions, for all properties, Table 1). At P7 we were able to compare both passive and active properties between the three groups. Though no difference were found between the three conditions (*P*>0.05 between the three conditions, for all properties, Table 1), all recorded neurons were capable of firing action potentials. Furthermore, dopaminergic neurons in slice preparation can be identified by characteristic hallmarks, such as the presence of the h-currents (Chu and Zhen, 2010; Grace and Onn, 1989). This current is reflected by the appearance of a hyperpolarizing ‘sag’ in the patch clamp recordings, as is the case at P7 in all three conditions (arrowheads, Fig. 2E). In addition, the majority of neurons (85%) displayed spontaneous action potential firing (Table 1). Thus, based on all included electrophysiological, active and passive properties (Table 1), eDA neurons are not dissimilar from control and En1KO mdDA neurons at E16.5 and P7.

**Table 1.**
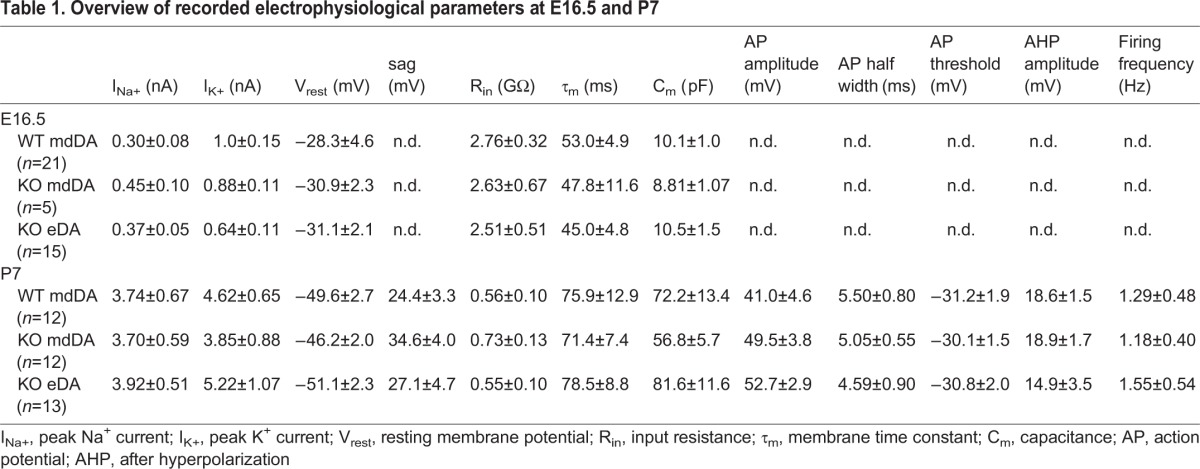
**Overview of recorded electrophysiological parameters at E16.5 and P7**

### The balance between dopaminergic and serotonergic neuron has shifted in the absence of En1

**Fig. 3.**
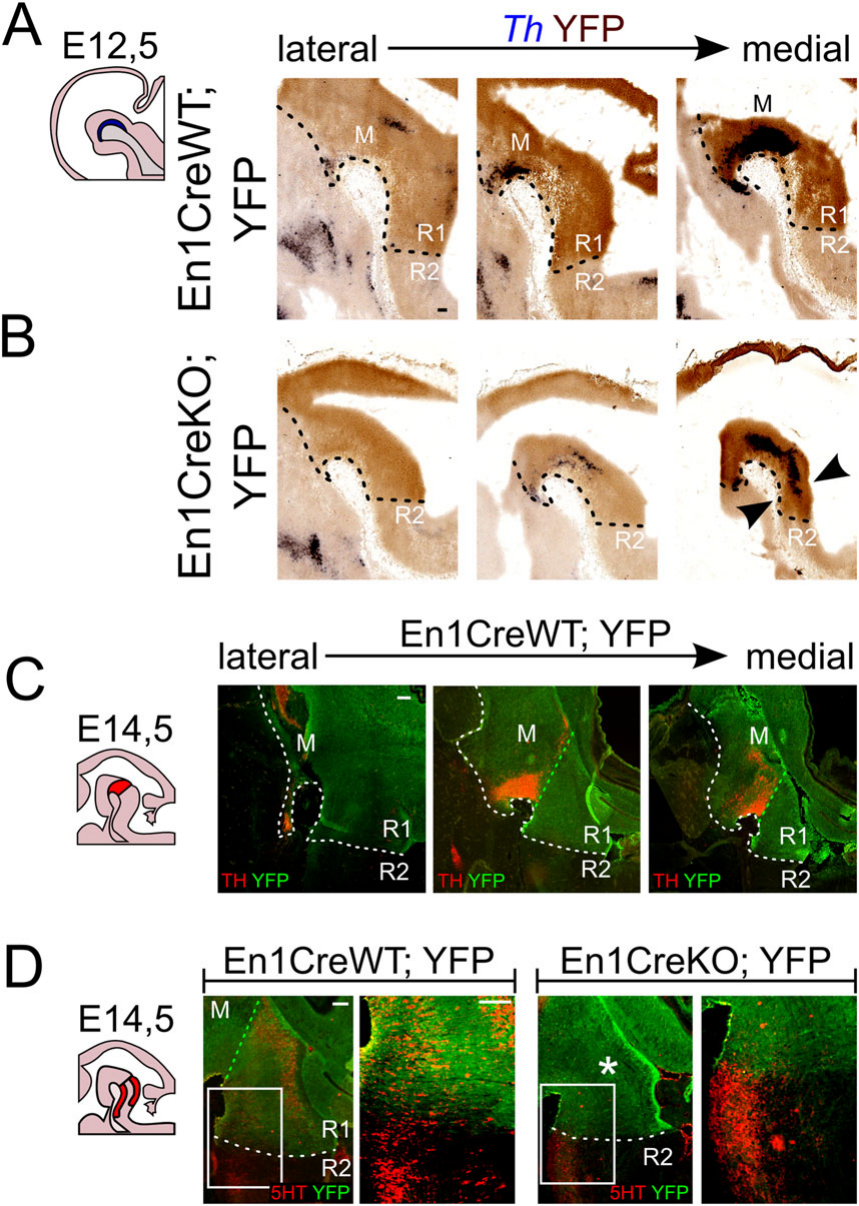
**Absence of En1 results in changes in the En1-derived R1 area, favoring dopaminergic neurons over serotonergic neurons.** (A,C) The region under the control of En1 extends rostrally to the border of dopaminergic neuronal generation in the ventral diencephalon and caudally to the presumed R1/R2 limit at E12.5. The black dotted line delineates the area that is under the control of En1, and therefore YFP-positive. (B) Ectopic DA neurons arise only in an YFP-positive, En1-derived area (arrowheads). (D) Midline section at E14.5 reveals that 5HT is lost in the En1-derived, YFP-area in *En1CreKO;YFP* (asterisk), but is still present caudal to the R1/R2-boundary. Area of higher magnification indicated by box in D; green dotted line represents the position of the isthmus; the white dotted line delineates the area that is under the control of En1, and therefore YFP-positive. M, midbrain; R1, rhombomere 1; R2, rhombomere 2. Scale bars: 100 μm.

It has previously been suggested that En1-derived cells make up the entire midbrain and R1 area (as visualized by *En1Cre;Wnt1-ΔMHB/+;R26dTomato* model in (Yang et al., 2013). In order to define the region that is under the control of En1 we lineage traced En1 using *En1^Cre/+^;R26RYFP/R26RYFP (En1CreWT;YFP)* and *En1^Cre/Cre^;R26RYFP/YFP* (*En1CreKO;YFP*) animals. In double labeling experiments at E12.5 we show that the region under the control of En1 extends to the ventral diencephalon and caudally to the presumed R1/R2 limit (Fig. 3A,C, indicated by the dashed line). Moreover, confirming our previous data, eDA neurons were generated in absence of En1 (*En1CreKO;YFP*) and are clearly confined to the region in which En1's influence is apparent (i.e. *Th*-expressing neurons are positioned in the YFP-positive area; Fig. 3B).

The appearance of dopaminergic neurons in rostral hindbrain (arrow head, Fig. 3B) hints towards a change in molecular coding in this region which might also influence the generation of 5-HT neurons. Analysis of *En1CreWT;YFP* and *En1CreKO;YFP* animals at E14.5 showed that 5-HT is absent in the region that is under the control of En1 (Fig. 3D, asterisk). Importantly, in the region caudal to the En1 limit (caudalwards from R2) 5-HT appears to be present similar to *En1CreWT;YFP* animals.

**Fig. 4.**
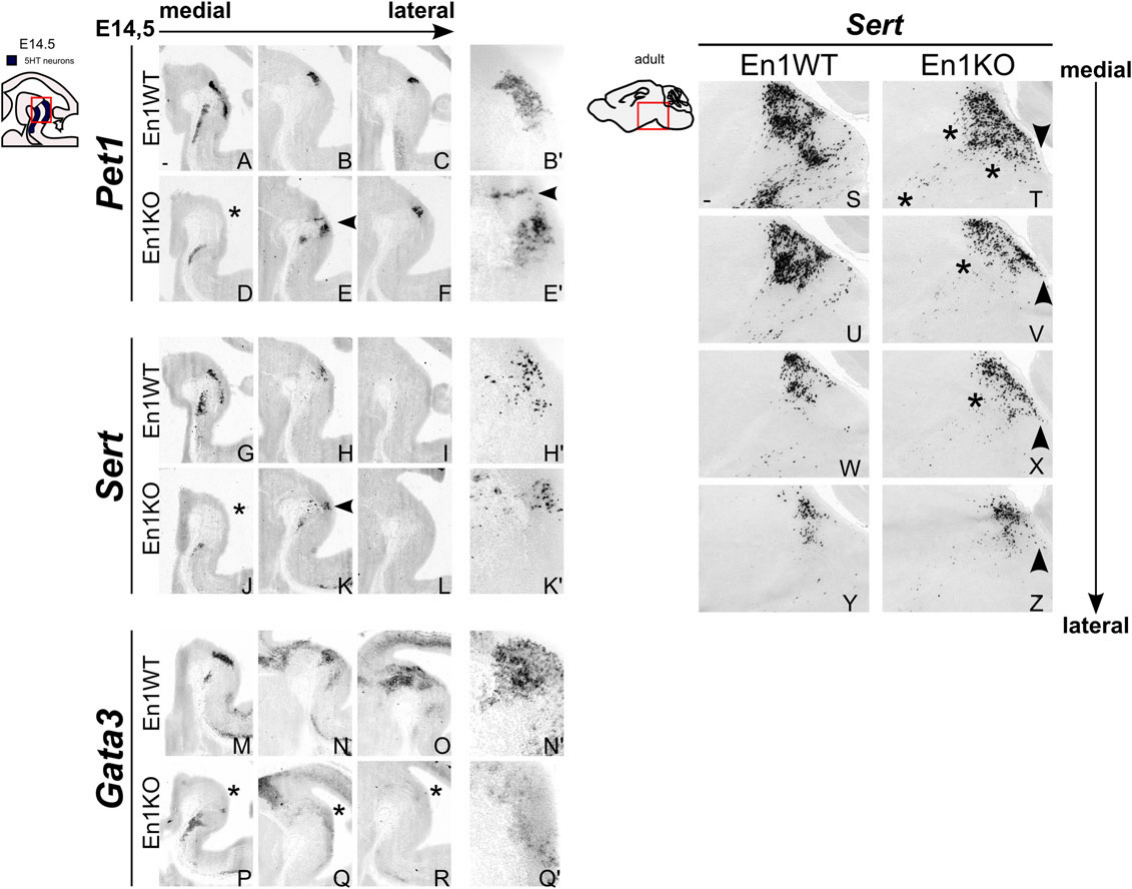
**R1-derived serotonergic system severely changed in absence of En1.** (A-R) At E14.5 all serotonergic markers investigated showed a severely changed expression pattern in absence of En1. *Pet1* (A-F), *Sert* (G-L) and *Gata3* (M-R) lost expression in DRN in medial section (asterisks). In paramedial sections the cytoarchitecture is changed (arrowheads) (B′,H′,N′,E′,K′,Q′). (S-Z) At P42 *Sert* expression is strongly diminished (asterisks) and the *Sert*-expressing neurons that were still present, are differently organized in En1KO brains compared to control (arrowheads). (Para)medial sections as shown in schematic, area shown indicated by a red box in schematic. Scale bars: 100 μm.

In order to further evaluate the loss of 5-HT neurons we investigated critical components of the molecular machinery essential for developing 5-HT neurons, such as *Gata3*, *Pet1 *and* Sert* at E14.5 (Deneris and Wyler, 2012). The serotonergic population can be divided (para-)medially into the dorsal raphe nucleus (DRN) (isthmic- or ventral R1-derived), the median raphe nucleus (MnR, ventral R1-derived) and the ventral prepontine raphe nucleus (PPnR, which is R2-derived) (Alonso et al., 2013). In the absence of En1 the expression of *Pet1*, *Sert* and *Gata3* was mostly lost in the medial DRN (asterisks in Fig. 4D,J,P), whereas in para-medial sections the DRN is still present, though differently organized (Fig. 4E′,K′,Q′, arrow). In agreement with the presence of 5HT in R2 in the En1CreKO (Fig. 3D), the expression of *Pet1*, *Sert* and *Gata3* seems unaffected in the PpnR, suggesting that at E14.5 the development of these serotonergic neurons progresses normally without En1 activity.

In order to establish that these prenatal changes were maintained into adulthood we analyzed *Sert* expression in adult control and En1KO animals (P42). In line with the data at embryonic stages, *Sert* expression was strongly diminished (Fig. 4S-Z, asterisks) and the *Sert*-expressing neurons that were still present, are differently organized in En1KO brains compared to control (Fig. 4S-Z, arrowheads). Together, the data derived from analysis at prenatal and adult stages suggest that the development of the ventral R1-derived region is malformed in the absence of En1, which is represented by a shifted balance in dopaminergic and serotonergic neuronal development.

### Absence of En1 leads to a disorganized IsO

The observed changed organization in the mid-hindbrain region suggests that the IsO may be defective as a consequence of En1 ablation, in addition to possible direct transcriptional defects in programming of dopaminergic and serotonergic neurons. During murine brain development the IsO (as defined by Fgf8 expression) is set at the juxtaposition of Otx2/Gbx2 expression at the rostral and caudal edge respectively (Crossley and Martin, 1995; Millet et al., 1999). This border is established at ∼E8 and continues to shape its surroundings until E12.5. In a conditionally En1-lineage tracing experiment using *En1^Cre-ERT/+^;R26RYFP/R26RYFP* animals (*En1Cre-ERT/WT;YFP*) we investigated the distribution of YFP-positive cells, when induced with Tamoxifen at E10.5. In the presence of En1 the Cre-induced YFP expression was restricted to the En1-positive cells at the IsO, forming a well-organized YFP-positive triangular area (Sgaier et al., 2005) surrounding the IsO at E14.5 (Fig. S3A,B). In contrast, the YFP-positive cells within the *En1Cre-ERT/KO;YFP* animals were sparsely found and not restricted to one location (Fig. S3C-C′). This is indicative of critical changes in the cytoarchitectural organization of the border between the midbrain and R1 in the absence of En1.

**Fig. 5.**
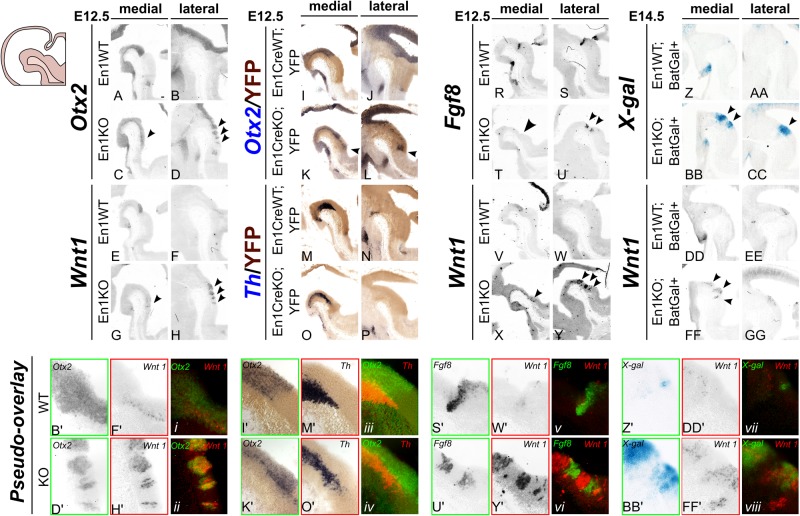
**In the absence of En1 the IsO is disorganized.** (A-D,I-L) *Otx2* expression is present in midbrain at E12.5, but is caudally extended into the hindbrain in En1KO and in En1CreKO, in a mosaic fashion (arrowheads). (E-H,V-Y) *Wnt1* expression is present in midbrain at E12.5, but is caudally extended into the hindbrain in En1KO, in a mosaic fashion (arrowheads). (i-ii) A pseudo-overlay reveals that the ectopic, mosaic expression of *Otx2* (green) and *Wnt1* (red) in En1KO is mutually overlapping. (M-P) *Th* expression is found ectopically in the YFP-positive R1 at E12.5 in En1CreKO in (para)medial sections. (iii-iv) The pseudo-overlay of *Otx2* (green) and *Th* (red) displays caudal ectopic expression in the En1CreKO. (R-U) *Fgf8* expression is present at the IsO in wild-type at E12.5, but is lost in medial sections in the En1KO and caudally multiplied in lateral sections (arrowheads). (v-vi) The pseudo-overlay of *Fgf8* (green) and *Wnt1* (red) displays caudal ectopic expression in the En1KO in a mutually exclusive manner. (Z-GG) β-galactosidase activity is present in IsO at E14.5 in *En1WT;BatGal+* animals, and is caudally extended in the *En1KO;BatGal+* (arrowheads in BB,CC). Adjacent sections of *Wnt1* mirror the β-galactosidase activity, both the restricted expression at the IsO in control and the enlarged area in *En1KO;BatGal*+ animals (arrowheads in FF). (vii-viii) The pseudo-overlay of β-galactosidase activity (green) and *Wnt1* (red) displays caudal and overlapping ectopic expression in the *En1KO;BatGal+*. Scale bar: 100 μm.

In order to understand which specific changes occur at the IsO in absence of En1, we investigated the expression of the pivotal isthmic determinants: *Otx2, Gbx2, Wnt1* and *Fgf8*. At E12.5 *Otx2* expression is normally restricted to the ventral midbrain (and more anterior brain regions) terminating at the IsO (Fig. 5A,B). Importantly in En1KO animals this expression is extended, far into the ventral hindbrain in a mosaic manner (Fig. 5C,D, arrowheads). The expression of Wnt1 is equally extended into the ventral hindbrain compared to controls (Fig. 5E-H, arrowheads). Interestingly, the extended pattern of *Otx2* and *Wnt1* expression is present in multiple stripes and this striate expression is overlapping (as suggested by the pseudo-overlay of adjacent sections in Fig. 5ii). In order to confirm whether the changes in *Otx2* expression in En1 mutants were restricted to the En1-derived ventral R1-area, we analyzed *Otx2* expression in *En1CreWT;YFP* and *En1CreKO;YFP* reporter animals. As expected, the ectopic expression of *Otx2* (and *Th* as a reference) at E12.5 is confined to the En1-expression domain (Fig. 5I,J,M,N). As described above, while in control sections *Otx2* expression is restricted at the IsO, the posterior limit in *En1CreKO;YFP* sections is shifted more caudally and now overlaps with the caudal border of the En1 expression domain (Fig. 5K,L, arrowheads).

In an independent set of experiments we analyzed *Fgf8* in order to confirm the loss of IsO integrity in the En1KO. Instead of one clear band of expression representing the IsO in control animals (Fig. 5R,S), the expression of *Fgf8* was lost in the most medial section whereas two separate bands of Fgf8 expression (of which the latter was positioned more caudally) were observed in the mutant (Fig. 5T,U). Adjacent sections analyzed for *Wnt1* expression reveal that the appearance of multiple bands of *Fgf8* expression are accompanied by a mosaic expression pattern of *Wnt1* (Fig. 5V-Y). Markedly, the pseudo-overlay of *Fgf8* and *Wnt1* reveals a mutual exclusive expression pattern (Fig. 5vi). Since *Fgf8* is considered to be present in the hindbrain-derived part of the IsO, whilst Wnt1 is only present in midbrain-derived tissue (Martinez et al., 1999), their mutual exclusive stripped pattern suggests that the *IsO* is fragmented in the En1-ablated mice. Notably, we investigated the expression of *Gbx2* to determine if the observed distorted IsO was influenced by aberrant expression of *Gbx2*. However, similar to control, the *Gbx2* expression was absent at E12.5 in En1KO animals, while adjacent sections with *Otx2* expression reveal a clear posterior shift (Fig. S4).

Since we observed over-expression of Wnt1 around the IsO in the En1KO (Fig. 5G,H,X,Y) we examined if the absence of En1 also resulted in changes in (canonical) Wnt-signaling. To do so, we generated *En1WT;BatGal+* and *En1KO;BatGal+* animals which serve as reporters for possible changed canonical Wnt signaling (Maretto et al., 2003; Mesman et al., 2014). In absence of En1 we find a strong upregulation of β-galactosidase activity, indicative for an upregulation of canonical Wnt signaling (Fig. 5BB,CC; for a complete data overview, including *Wnt1* and *Th* expression as reference see Fig. S5). Thus, in contrast to control animals (Fig. 5Z,AA), in the absence of En1, both *Wnt1* expression (Fig. 5DD-GG) and canonical Wnt-signaling are markedly upregulated at E14.5. To conclude, our data indicate that En1 is essential for the (direct or indirect) repression of *Wnt1* and *Otx2* in R1, and establishment or maintenance of appropriate *Fgf8* expression, which together may form the molecular basis of a disturbed IsO in the absence of En1.

## DISCUSSION

### Engrailed 1 is essential for proper IsO development and function

During normal murine brain development the IsO (as defined by Fgf8 expression) is set at the juxtaposition of Otx2/Gbx2 expression at ∼E7-8 (Crossley and Martin, 1995; Martinez et al., 1999; Millet et al., 1999). The requirements for proper IsO development change dynamically over time; the classic removal of Gbx2 results in an expanded expression of Otx2 and a caudal relocation of the IsO (Wassarman et al., 1997), whereas the IsO region develops normally, when Gbx2 is conditionally removed after ∼E9 (Li et al., 2002). Furthermore, at E12.5 the linear expression of *Fgf8* marks the caudal limit of Otx2 expression as a boundary between midbrain and R1, although *Gbx2* is no longer present in R1 (Fig. S4). Consequently, it has been suggested that a Gbx2-independent route is required to repress Otx2 in the metencephalon after ∼E9 (Li et al., 2002).

In the full En1KO we observed a (mosaic) expansion of *Otx2* expression at E12.5, which resulted in a fragmentation of the IsO as is marked by ectopic *Fgf8* expression (Fig. 5). Strikingly, the altered expression of *Otx2* in the En1KO was mimicked by the identical expression of *Wnt1* (Fig. 5ii), whilst fragmented *Fgf8* expression revealed to be mutually exclusive to the *Wnt1* expression (Fig. 5vi). These data are in line with previous research on overexpression of Otx2 and Wnt1 in midbrain and R1. The single overexpression of Otx2 resulted in the ectopic presence of Wnt1 and vice versa, and both mutant models induced dopaminergic neurons in the rostral hindbrain (Brodski et al., 2003; Prakash et al., 2006). Moreover, our work shows that the expansion of Wnt1 expression was accompanied by a similar expansion of canonical Wnt-signaling (Fig. 5viii, Fig. S5). Forced expression of β-catenin results in a similar phenotype as observed here: Th-positive neurons emerge in rostral R1 (Joksimovic et al., 2012). In other words, the current En1-null mouse reveals strong similarities with research approaches that conditionally over-express Wnt1, Otx2 or β-catenin (Brodski et al., 2003; Joksimovic et al., 2012; Prakash et al., 2006), and all approaches lead to an extension of the mesencephalon, including the appearance more caudally located Th-expressing neurons. Together, our data suggest that in absence of En1 the mesencephalon area is posteriorly extended, at the expense of the ventral R1 region. Interestingly, since the cerebellum is derived from the dorsal R1 (Wingate, 2001; Wingate and Hatten, 1999), its presence in the viable En1KO (on C57BL6/J background) suggests that (at least part of) dorsal R1 develops normally.

To recapitulate, these studies and our data suggest that the changed dynamics of the IsO in absence of En1 are primarily due to misexpression of Otx2 and Wnt1, resulting in the appearance of eDA neurons at the expense of 5HT neurons as a secondary consequence. In this sense, En1 might fulfill a role that is very similar to that of Gbx2, i.e. the repression of Otx2. Evidently, Gbx2 represses Otx2 earlier in development, however after ∼E9 Gbx2 is no longer required (Li et al., 2002), which just coincides with the time point of the expression of En1 in the region. This notion is supported by the observation that the phenotype of the En1KO highly resembles the Gbx2KO (Wassarman et al., 1997). We propose that En1 is essential for continued repression of Otx2 thereby enabling a 5-HT phenotype in R1.

### A general role for En1 in boundary formation?

In support of our current work that reveals that En1 is essential for proper IsO maintenance and function, other groups have shown that En1 fulfills a similar role in limb development. En1KO animals display limb malformations such as polydactyly and a double-dorsal paw (i.e. the ventral paw becomes hairy and pigmented), independent of the genetic background of the mutant line (Adamska et al., 2004; Bilovocky et al., 2003; Wurst et al., 1994). This phenotype is explained by an ectopic or secondary apical ectodermal ridge (AER), a signaling center similar to the IsO. The AER forms an Fgf8-expressing border between ventral ectodermal tissue and dorsal ectodermal tissue during limb formation (Moon and Capecchi, 2000). These changes in AER development are accompanied by the ectopic expression of Wnt7a and Wnt-signaling (Adamska et al., 2004; Cygan et al., 1997; Loomis et al., 1998). To recapitulate, in En1-ablated animals both the AER and the IsO are characterized by ectopic expression of Fgf8, Wnts and canonical Wnt-signaling, resulting in the expansion of dorsal ectoderm and midbrain, which ultimately leads to polydactyly as well as dopaminergic neurons in the metencephalon.

### How does En1 support a dopaminergic cell fate in midbrain but suppress it in R1?

En1 is present in midbrain and R1, its expression spans the IsO. This introduces the question via which mechanism En1 induces dopaminergic neurons rostrally and serotonergic neurons caudally of the IsO? In drosophila, En exerts a repressive effect on its targets in the absence of Exd, whilst in the presence of Exd, En activates its targets (Serrano and Maschat, 1998). The murine orthologue of Exd is Pbx1, and if its relationship to En1 in mouse is similar to the relationship between Exd and En in drosophila, the differential presence of Pbx1 in midbrain (but not R1) might play a decisive role in determining the activator role of En1 on its targets in midbrain (but not R1). Research on the Pbx1KO reveal that mdDA neurons develop a normal molecular profile (though neurons display disrupted axon guidance) (Sgadò et al., 2006). However, the possibly compensatory presence of Pbx3, might cloak the true regulatory role of Pbx1 on En1 function. Secondly, in an impressive double publication Kurokawa and colleagues show a repressive regulator mechanism controlling Otx2 expression (Inoue et al., 2012; Kurokawa et al., 2004a,b). Otx2 contains an enhancer region 115 kb 3′ downstream (the X29 sequence) which induces Otx2 expression activities in midbrain from E8.5 onwards. The authors propose that the TAATTA sequence within X29 is recognized by Brn1/2/4 and Oct6 in midbrain leading to activation of Otx2, whereas Gbx2 competes for binding to the same sequence in hindbrain and successfully represses Otx2. Interestingly, En1 is similarly known to recognize TAATTA (Draganescu and Tullius, 1998), and thus the repressive role of En1 on Otx2 in R1 possibly occurs through to the X29 regulatory sequence.

### Concluding remarks

In the current manuscript we elected to investigate the role of En1 in the viable En1-null mouse (the *En1^tm1Alj/+^* animals back-crossed to the C57BL6/J line; Bilovocky et al., 2003; Veenvliet et al., 2013) in order to link developmental mechanisms of mid-hindbrain formation to the adult landscape of dopaminergic and serotonergic neurons. Our work suggests that En1 controls the repression of Otx2, Wnt1 and canonical Wnt-signaling in ventral R1. Ablation of En1 changes the patterning around the IsO and induces properly coded and functional eDA neurons at the expense of serotonin neurons. This suggests that En1 is pivotal to IsO maintenance and function.

## MATERIALS AND METHODS

### Animals

Embryos from several mouse lines were isolated at embryonic day (E)12.5, E14.5, and E16.5, considering the morning of detection of the vaginal plug as E0.5. Tissue was isolated at postnatal day (P)0 (day of birth), P7, P42 and one year after birth.

Several mutant mouse lines were used during this study; all of them were back-crossed to the C57BL6/J line. First, *En1^tm1Alj/+^* animals were back crossed to the C57BL6/J line generating *En1^+/+^* (WT), *En1^tm1Alj/+^* (Het) and viable *En1^tm1Alj/tm1Alj^* (KO) offspring (previously described in Bilovocky et al., 2003; Veenvliet et al., 2013). Second, *En1^tm1Alj/+^* animals were inter-crossed with *Pitx3^gfp/gfp^* animals in which the Pitx3 gene is substituted by a GFP allele (Jacobs et al., 2011) in order to breed *En1^+/+^;Pitx3^gfp/+^* (*En1WT;Pitx3GFP/+*) and viable *En1^tm1Alj/tm1Alj^;Pitx3^gfp/+^* litter mates (*En1KO;Pitx3GFP/+*)*.* Third, *En1^tm1Alj/+^* animals were inter-crossed with the transgenic mouse line *B6.Cg-Tg(BAT-lacZ)3Picc/J* (BAT-GAL) (Maretto et al., 2003), in order to generate *En1^+/+^;BatGal/+* (*En1WT;BatGal/+*) and *En1^tm1Alj/tm1Alj^;BatGal/+* (*En1KO;BatGal/+*) litter mates . Fourth, *En1^Cre/+^* animals were inter-crossed with *En1^Cre-ERT+/+^;R26RYFP/R26RYFP* (Sgaier et al., 2005) to generate *En1^Cre/+^;R26RYFP/+* animals. We back-crossed these animals, in order to study the following genotypes: *En1^Cre/+^;R26RYFP/R26RYFP* (*En1CreWT;YFP*) and *En1^Cre/Cre^;R26RYFP/R26RYFP* (*En1CreKO;YFP*). In these animals YFP is expressed continuous in the En1 expression region; i.e. the midbrain and R1. Fifth, *En1^Cre-ERT/+^;R26RYFP/R26RYFP* (Sgaier et al., 2005) were also back-crossed with *En1^tm1Alj/+^* animals to enable En1- lineage tracing by induction of Cre-ERT, through oral administration (using a gavage) of Tamoxifen (Sigma) 20 mg/ml in corn oil (Sigma), at different time points (E10.5). When Tamoxifen was administered at E10.5 embryos were isolated at E14.5.

All animals were genotyped by PCR using specific primers (Table 2). *Pitx3^gfp/gfp^* animals were recognized by the shape of the lens, which is malformed in all Pitx3-deficient animals. All procedures and experiments were performed according to the guidelines and with the approval of the Dutch Ethical Committee of the University of Amsterdam.

**Table 2.**
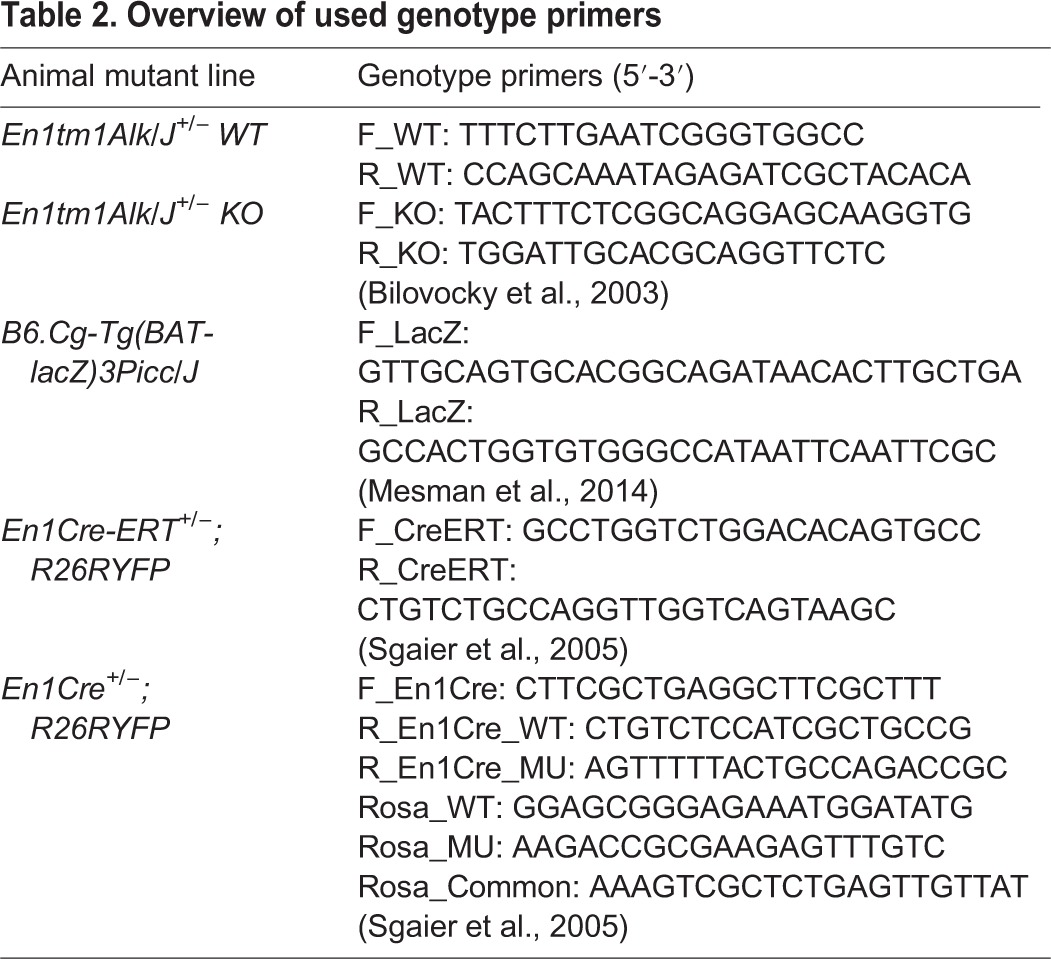
**Overview of used genotype primers**

### *In situ* hybridization (ISH)

*In situ* hybridization was performed as described previously (Smits et al., 2003). Digoxigenin-labeled probes for *Th*, *En2*, *Vmat2*, *Dat*, *Nurr1*, *AADC*, *Lmx1b*, *Pitx3*, *Pbx1*, *Pbx3* and *Wnt1* were used as previously described (Grima et al., 1985; Hoekstra et al., 2012; Mesman et al., 2014; Smidt et al., 2000a,b; Veenvliet et al., 2013). Additional probes: *Fgf8* (500-1003 bp, NM_010205), *Otx2* (NM 144841.2, bp 592-1165), *Sert* (bp 1827-2326, NM_010484.1), *Gata3* (bp 1312-1685, NM008091.3), *Pet1* (bp 885-1444, NM_153111.2), *Gbx2* (bp 777-1199, NM_010262).

### Fluorescence immunohistochemistry

Embryos were fixed in 4% paraformaldehyde (PFA) in PBS, cryoprotected in 30% sucrose in PBS and subsequently stored at −80°C. Sagittal sections (16 µm) were cut on a cryostat, after which they were washed with TBS and blocked in 4% fetal calf serum (FCS) in THZT (50 mM Tris-HCl pH 7.6, 0.5 M NaCl, 0.5% Triton X-100). After another wash treatment with TBS, sections were incubated overnight at 4°C with primary antibody in THZT. Sections were washed three times (TBS) the following morning and incubated for minimally 2 h at room temperature (RT) with secondary antibody in TBS, followed by wash treatment with PBS. Finally sections were embedded with Fluorsave (Biochemical). Primary antibodies that were used: Rabbit α-Th (Pelfreeze, 1:1000), Rabbit α-serotonin (ImmunoStar, 1:500), Rabbit α-Pitx3 (Smidt et al., 1997; 1:500), Chicken α-GFP (Abcam, 1:1000). Sheep-α-GFP (Biogenesis, 1:500). Secondary antibodies that were used: Goat α-Rb Alexa Fluor 555 (1:1000), Goat α-Rb Alexa Fluor 488 (1:1000), Goat α-Chicken Alexa Fluor 488 (1:1000), all from Invitrogen.

### X-galactosidase staining protocol

Fresh frozen sections were defrosted and post-fixed with 4% PFA for 45 min. Sections were washed with PBS three times, and once with staining solution (5 mM potassium ferricyanide, 5 mM potassium ferrocyanide, 2 mM MgCl_2_ in PBS). Sections were incubated at RT with staining solution, complemented with 1 mg/ml X-galactosidase (X-gal) for several hours, protected from light, until staining was optimal. Sections were then rinsed in PBS, dehydrated and embedded in Entellan (Merck).

### Electrophysiology recordings

For recordings at E16.5, pregnant dams were sacrificed by cervical dislocation and embryos were isolated from the uterus. Neural tubes were micro-dissected on ice in 5% FCS Leibovitz-15 (Sigma), the telencephalon was removed and the remaining neural tube was cut along the medial axis. The resulting ‘hemitubes’ were subsequently used for recordings at the medial plane. The eDA neurons are discriminated from mdDA neurons in the En1-mutant based on their ectopic (caudal) location in the hindbrain.

For recordings at P7, brains were isolated in ice-cold artificial cerebrospinal fluid (ACSF) containing (in mM): NaCl (120), KCl (3.5), CaCl_2_ (2.5), MgSO_4_ (1.3), NaH_2_PO_4_ (1.25), NaHCO_3_ (25), glucose (25), continuously bubbled with 95% O_2_/5% CO_2_ (pH 7.4) and 250 µm-thick saggital slices were cut on a vibroslicer (Leica VT1000S). Hemitubes and slices were transferred to a recording chamber and continuously superfused with ACSF. Patch pipettes were pulled from borosilicate glass and had a resistance of 4-6 MΩ when filled with internal solution containing (in mM): potassium gluconate (105), KCl (30), EGTA (5), CaCl_2_ (0.5), HEPES (10), and Mg-ATP (5) (pH 7.3 with KOH). GFP-positive neurons were visualized using differential interference contrast microscopy on a Zeiss FS2 microscope equipped with standard epiﬂuorescence. Whole-cell recordings were made using an EPC9 patch-clamp ampliﬁer and PULSE software (HEKA Electronik, Lambrecht, Germany). Cells were voltage clamped at −70 mV (corrected for liquid junction potential) and series resistance was compensated for at least 70%. Signals were ﬁltered at 1-5 kHz, sampled at 10 kHz, and off-line analysis was performed using Igor Pro (Wavemetrics, Lake Oswego, OR, USA).

### Statistical analysis

Values of electrophysiological measurements are expressed as means±standard error of the mean (s.e.m.). Comparisons were made using two-tailed Student's *t*-test. *P*<0.05 was considered significant, and indicated using an asterisk (*).
